# Climate change and health: Moving from theory to practice

**DOI:** 10.1371/journal.pmed.1002628

**Published:** 2018-07-31

**Authors:** Jonathan A. Patz, Madeleine C. Thomson

**Affiliations:** 1 Global Health Institute, University of Wisconsin, Madison, Wisconsin, United States of America; 2 Center for Sustainability and the Global Environment (SAGE), Nelson Institute for Environmental Studies, University of Wisconsin, Madison, Wisconsin, United States of America; 3 Department of Population Health Sciences, University of Wisconsin, Madison, Wisconsin, United States of America; 4 International Research Institute for Climate and Society (IRI), Columbia University, Palisades, New York, United States of America; 5 Department of Environmental Health Sciences, Mailman School of Public Health, Columbia University, New York, New York, United States of America

## Abstract

In an Editorial discussing the Special Issue on Climate Change and Health, guest editors Jonathan Patz and Madeleine Thompson summarize key issues in the field and describe the significance of research studies included in the issue.

## Background

This year marks the 30th anniversary of the Intergovernmental Panel on Climate Change (IPCC), which has produced five comprehensive assessments for the world’s governments; a health chapter has featured in all but the first assessment report, along with climate change impacts on our atmosphere, oceans, and landscapes. Research on the health risks from climate change has grown substantially, with findings suggesting that the global health gains achieved over the past half century are being undermined by climate change [1]. Hazardous exposure pathways are many, from heat waves and air pollution episodes to infectious diseases, malnutrition, forced migration, and conflict [2]. Impacts are experienced differently within segments of the population and between geographic locations based on biological, social, and economic vulnerabilities as well as the nature of the climate hazard.

## Health implications of climate change

This special issue of *PLOS Medicine* brings together the latest research across many of these health risk pathways, as well as highlighting at-risk populations, such as women [3], children [4], and small island populations [5]. New findings indicate that heat waves can affect cognitive abilities [6], rainfall extremes could increase sewage contamination in cities [7], warmer temperatures produce hazardous ozone air pollution [8], and wildfires, as occurred recently in California, have substantial adverse consequences for human health [9]. Some surprising findings from research studies have also emerged: countering the perceived potential benefit of carbon dioxide (CO_2_) emissions on plant growth and food security [10], CO_2_-induced nutritional deficiencies have been highlighted [11].

The 2015 Paris Climate Agreement marked a point in time when world leaders came together and recognized that climate change is real, is already occurring, and that action and investment to mitigate climate change are urgently required [12]. Ironically, the actions taken to grapple with climate change may represent some of the largest opportunities in more than a century to advance global public health [13]. Consider the following: first, greenhouse gas (GHG) emissions have continued to rise from 1970 to 2010, with the largest increases between 2000 and 2010, despite a growing number of climate change mitigation policies [14]; second, the World Health Organization (WHO) estimates that 7 million premature deaths annually are attributed to the consequences of air pollution [15], much of it produced in association with GHG emissions; and finally, rates of obesity and chronic diseases are rising in nearly all regions of the world, and an estimated 5.3 million premature deaths each year are attributed to sedentary lifestyles supported by fossil fuel-intensive transport [16].

Thus, cleaner energy can both reduce climate change and simultaneously save lives endangered by air pollution. In addition, obesity, diabetes, heart disease, and cancer, which are in part related to physical inactivity [17], may be reduced by a switch to low-carbon transport—including walking and cycling. Furthermore, the health benefits of clean air may far outweigh the investment costs of clean energy technologies, providing additional economic incentives for mitigation activities [18]. In the United States, a life cycle analysis found that the health benefits of a national low-carbon energy plan would offset energy investment costs by between 26% and 1,050% [19]. In *PLOS Medicine*, Woodcock and colleagues estimate the health and economic benefits that could accrue from increasing the number of cyclists in the United Kingdom, and provide a decision support tool for local health officials and urban planners tasked with preparing the built environment for healthier communities [20].

## Climate change: Projections and planning

While not new, the idea that, in addition to food choices and nutrition, energy choices and transportation planning relate to human health is a reminder of the importance of the Health in all Policies (HiAP) approach promoted by WHO [21]. The collective body of new research presented in this special issue of *PLOS Medicine* reinforces the need for such a systems-based approach; clearly, policies applied to electric power generation, transportation planning, and food systems must be regarded as public health policies. This holds both for the risks from and potential solutions to climate change.

However, no matter how quickly the world can reduce carbon emissions, inertia in the earth’s climate system commits us to some extent to endure the consequences of ongoing anthropogenic climate change. Adapting to our changing climate is essential if hard-won health gains are not to be lost to sea level rise and a warmer, more extreme climate. Modelling the future climate has been essential to the development of climate change mitigation strategies, and adaptation strategies are also dependent on some advance knowledge of the future climate. One challenge for decision-makers is that while mitigation is a global phenomenon (reducing carbon emissions in any part of the world would have a collective benefit for everyone), adaptation is a local necessity. Health decision-makers must understand specific health consequences of responses to climate change, as experienced by beneficiaries of the local health system, often at the district level or below. And climate is complex. It behaves differently according to climate region and local geography, and varies by spatial distance and on multiple timescales. The current mixing of climate variability and signals of longer-term change [22] adds further uncertainties to the known limits of climate change models to predict rainfall and temperature at the local level over the coming decades.

New studies in this issue of *PLOS Medicine* also show interlinkages and trade-offs between interventions for adaptation and mitigation. For example, Achebak and colleagues show that mortality during summer in Spain is declining in the face of an increasing number of heat waves [23]. The presumption is that technologies or adaptive behavior are advancing and making people less likely to die during a heat wave. But a study focused on the US [24] shows the potential for increased usage of air conditioning to cause additional morbidity and mortality if electricity is still generated in part from coal-fired power plants. In addition, pursuing a low-carbon economy will require vigilance to ensure that some populations are not disadvantaged, as Cushing and colleagues report on air-pollution inequities emerging from a carbon-pricing policy in California [25]. These new studies highlight potential unintended consequences of both adaptation and mitigation measures and require us to take a systems approach to every intervention to avoid trading one health problem for another.

Risks from climate change are now mainstream in the health discourse. For example, the subject captured the annual conference theme, “Climate Changes Health,” for the American Public Health Association (APHA) in 2017. Large health and medical coalitions have formed, such as the Global Climate and Health Alliance (http://www.climateandhealthalliance.org/), the Medical Society Consortium on Climate and Health (https://medsocietiesforclimatehealth.org/), and Global Green and Healthy Hospitals (a network of Health Care Without Harm, https://noharm-global.org/issues/global/global-green-and-healthy-hospitals), to name a few. The healthcare system has even been examining its own contribution to GHG emissions, as Eckelman and colleagues analyzed in the case of Canada in this issue [26]. New funding streams have come online, such as the “Our Planet our Health” programme of the UK’s Wellcome Trust [27], the first major biomedical foundation to specifically engage about climate change. The Belmont Forum, a consortium of 26 major and emerging funding agencies concerned with global environmental change, has signaled its interest in investing in research on health and climate change.

Major initiatives to reduce the health consequences of air pollution and climate change are primarily focused on rich countries or emerging economies, where public concern has been greatest, the economic benefits of mitigation and adaptation more readily quantified, and the resources needed to change behavior more readily accessible. The Green Climate Fund, created specifically to serve low- and middle-income countries, has as yet received few successful applications that focus on health. According to WHO, "less than 1.5% of international finance for climate change adaptation is allocated to projects which ensure that the health of all people is preserved, and only a fraction of this supports small island developing states” [28], and the plight of Small Island Developing States (SIDS) highlighted under the new climate change strategy of WHO prioritizes the most vulnerable communities while fostering innovation and health systems strengthening [29].

## Conclusion

In summary, multiple lines of evidence and research have shown climate change to be a threat to global health. At the same time, actions targeting the cause of climate change (reducing GHG emissions) offer large health benefits, especially in the area of noncommunicable diseases (NCDs). The challenge is now to bring interventions to scale—practical action requires an informed health workforce, an engaged public, an HiAP approach involving many related sectors, new resources and new technologies, and financing equal to the task at hand. Cross-sector indicators by which to measure progress have been proposed [30]. With the falling price of clean energy technologies, economic forces are already steering society toward a low-carbon future [31]. However, the pace must be rapidly accelerated to assure the future health and well-being of populations across the globe, prioritizing the most vulnerable communities not only in high-income countries but also in low- and middle-income countries. To achieve this acceleration, tailored resources that can be used in teaching climate change and health must be developed and integrated into the core curricula of public health physicians, nurses and other health workers as a priority.

## Abbreviations

APHA: American Public Health Association
GHG: greenhouse gas
HiAP: Health in All Policies
IPCC: Intergovernmental Panel on Climate Change
NCD: noncommunicable diseases
SIDS: Small Island Developing States
WHO: World Health Organization
